# Concrescence in anterior teeth assessed by cone beam computed tomography. A case report

**DOI:** 10.21142/2523-2754-1001-2022-102

**Published:** 2022-03-30

**Authors:** David Aristizábal-Elejalde, Juan Alejandro Casanova-Sarmiento, María Eugenia Guerrero, Aron Aliaga-del Castillo, Gustavo Armando Ruiz-Mora, Yalil Augusto Rodríguez-Cárdenas

**Affiliations:** 1 Division of Oral and Maxillofacial Radiology, School of Dentistry, Universidad Científica del Sur, Lima, Perú. davidaristizabal4@hotmail.com Universidad Científica del Sur Division of Oral and Maxillofacial Radiology School of Dentistry Universidad Científica del Sur Lima Peru davidaristizabal4@hotmail.com; 2 Division of Oral and Maxillofacial Radiology, School of Dentistry, Universidad Científica del Sur, Lima, Perú. Juancasanova1192@gmail.com Universidad Científica del Sur Division of Oral and Maxillofacial Radiology School of Dentistry Universidad Científica del Sur Lima Peru Juancasanova1192@gmail.com; 3 Division of Oral and Maxillofacial Radiology, School of Dentistry, Universidad Nacional Mayor de San MarcosLima, Perú. mega4343@yahoo.com.mx Universidad Nacional Mayor de San Marcos Division of Oral and Maxillofacial Radiology School of Dentistry Universidad Nacional Mayor de San Marcos Lima Peru mega4343@yahoo.com.mx; 4 Department of Orthodontics. Bauru Dental School. University of Sao Paulo, Brazil. a_aliaga@hotmail.com Universidade de São Paulo Department of Orthodontics Bauru Dental School University of Sao Paulo Brazil a_aliaga@hotmail.com; 5 Division of Orthodontics, Faculty of Dentistry, Universidad Nacional de Colombia, Bogotá D.C, Colombia. garruiz@gmail.com Universidad Nacional de Colombia Division of Orthodontics Faculty of Dentistry Universidad Nacional de Colombia Bogotá D.C Colombia garruiz@gmail.com; 6 Division of Oral and Maxillofacial Radiology, Faculty of Dentistry, Universidad Nacional de Colombia, Bogotá D.C, Colombia. yalilrodriguez@gmail.com Universidad Nacional de Colombia Division of Oral and Maxillofacial Radiology Faculty of Dentistry Universidad Nacional de Colombia Bogotá D.C Colombia yalilrodriguez@gmail.com

**Keywords:** dental concrescence, dental abnormalities, cone beam computed tomography, concrescencia dental, anomalías dentales, tomografía computarizada de haz cónico

## Abstract

Dental concrescence is an anomaly in which the cementum overlying the roots joins, causing the union of two different teeth. It is often reported in posterior dentition, affecting certain dental procedures such as root canal treatment, periodontal procedures, orthodontic movement and dental extraction. This case report describes the successful diagnosis and treatment of a 20-year-old male with a moderate skeletal class II who was referred for a radiographic evaluation after 1 year of failed orthodontic movement of teeth 1.1 and 1.2. The radiographic assessment with a Cone Beam Computed Tomography allowed discard other related pathologies and diagnose a dental concrescence. The patient underwent orthognathic surgery in which the class II was corrected, and the concrescence was treated with a prosthetic approach.

## RESUMEN

La concrescencia dental es una anomalía en la que el cemento que recubre las raíces se une, y provoca la unión de dos dientes diferentes. A menudo, se presenta en la dentición posterior y afecta ciertos procedimientos dentales, como el tratamiento del conducto radicular, los procedimientos periodontales, el movimiento de ortodoncia y la extracción dental. Este informe de caso describe el diagnóstico y tratamiento exitoso de un hombre de 20 años con una clase II esquelética moderada, que fue remitido para una evaluación radiográfica después de 1 año de movimiento ortodóncico fallido de los dientes 1.1 y 1.2. La valoración radiográfica con una tomografía computarizada de haz cónico permitió descartar otras patologías relacionadas y diagnosticar una concrescencia dentaria. El paciente fue sometido a una cirugía ortognática en la que se corrigió la clase II y se trató la concrescencia con abordaje protésico.

## INTRODUCTION

Dental concrescence is a dental anomaly classed as a type of alteration. In this anomaly, two dental roots are united by the cement tissue, but the dental crowns remain separated. There are two different types of dental concrescence: “congenital” and “acquired”. The former originates during dental formation, and the latter occurs after the apical formation ends. Inflammatory responses are the main cause by which a dental concrescence is acquired. In this type, the close distance to the roots causes an interdental bone resorption that joins both dental roots via dental cement apposition. The union of the roots can be partial or total ^1^^,^^2^.

**Figure 1:**
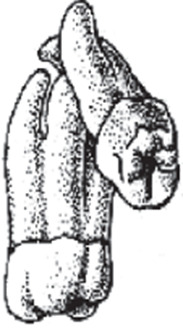
Concrescence in posterior teeth.

The majority of reported cases are in the posterior dentition, with the maxillary molars being the most affected teeth (Figure 1), particularly the third molars with a supernumerary tooth ^3^. Studies have reported that 0.8% of permanent molars that are extracted have this condition, and the prevalence is not related to age, sex or race. Special care should be given to ensure a correct diagnosis for this condition to avoid complications and to inform patients about the dental condition and risks and limitations of the treatment ^4^.

The presence of dental concrescence can alter the outcome of surgical, periodontal, endodontic and orthodontic treatments ^5^^,^^6^. When this dental anomaly is present, an interdisciplinary approach should be performed to prevent or overcome complications during dental treatment to help provide the patient with a functional and pleasant smile ^7^.

Dental concrescence can be treated with different approaches, including dental sectioning ^2^^,^^5^, dental extraction ^3^, or in a more conservative approach, restorative camouflage ^7^. When using bidimensional images, the assessment of dental concrescence becomes difficult due to the technical limitations that conventional radiography carries, such as image distortion and image superimposition ^8^. This latter condition complicates the images of the periodontal tissues surrounding the dental root. Currently, due to the easy access to tridimensional images, most of these limitations can be overcome. Tomography allows clinicians to evaluate each millimeter surrounding the root and verify whether the interdental bone space is absent ^9^.

**Figure 2:**
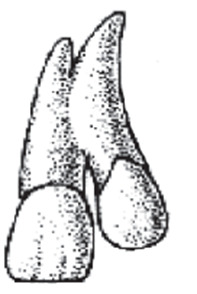
Concrescence in anterior teeth.

It is unlikely to find dental concrescence in adult patients where the anterior teeth are affected (Figure 2) ^10^. The objective of this case report is to provide information about the presence of dental concrescence between the maxillary incisors. Here, the patient underwent orthodontic treatment to try and correct the position of these teeth for almost a year without a proper response. A tridimensional analysis was performed to establish a diagnosis.

## CASE REPORT

**Figure 3:**
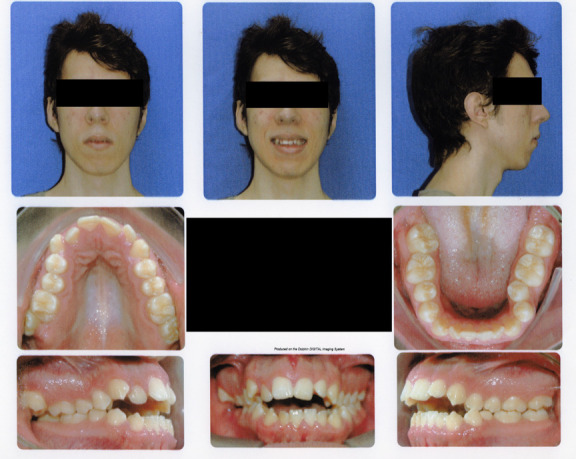
Pre-treatment facial and intraoral photographs.

**Figure 4:**
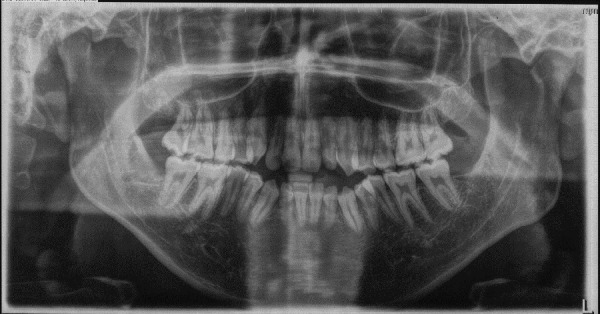
Pre-treatment panoramic radiograph.

**Figure 5:**
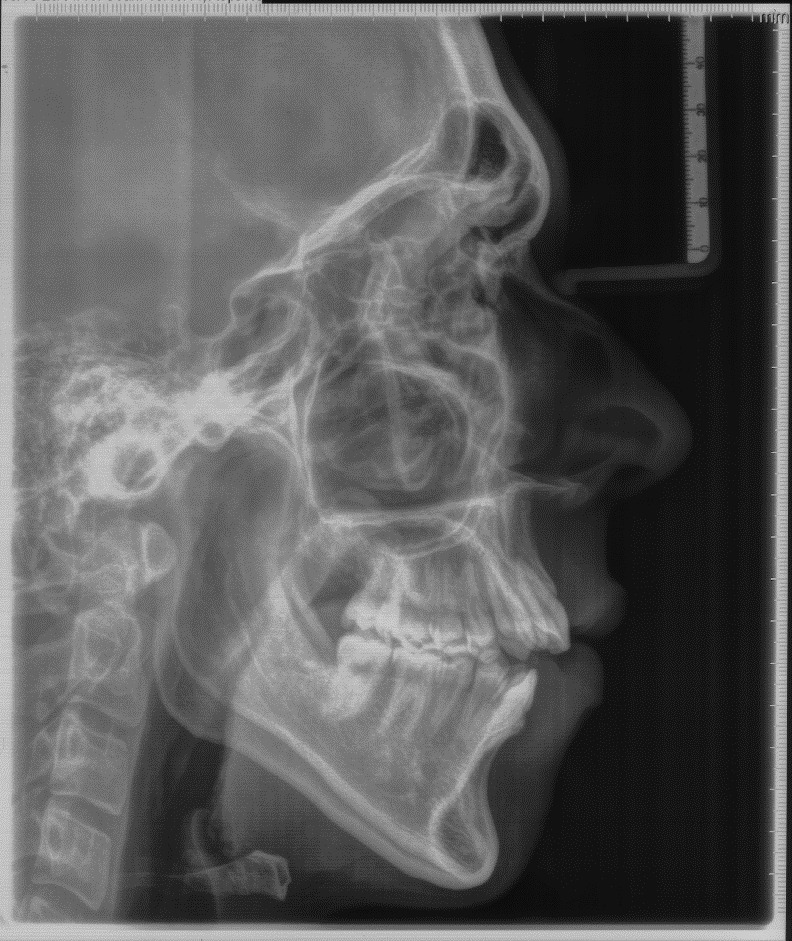
Pre-treatment lateral cephalometric radiograph.

**Figure 6:**
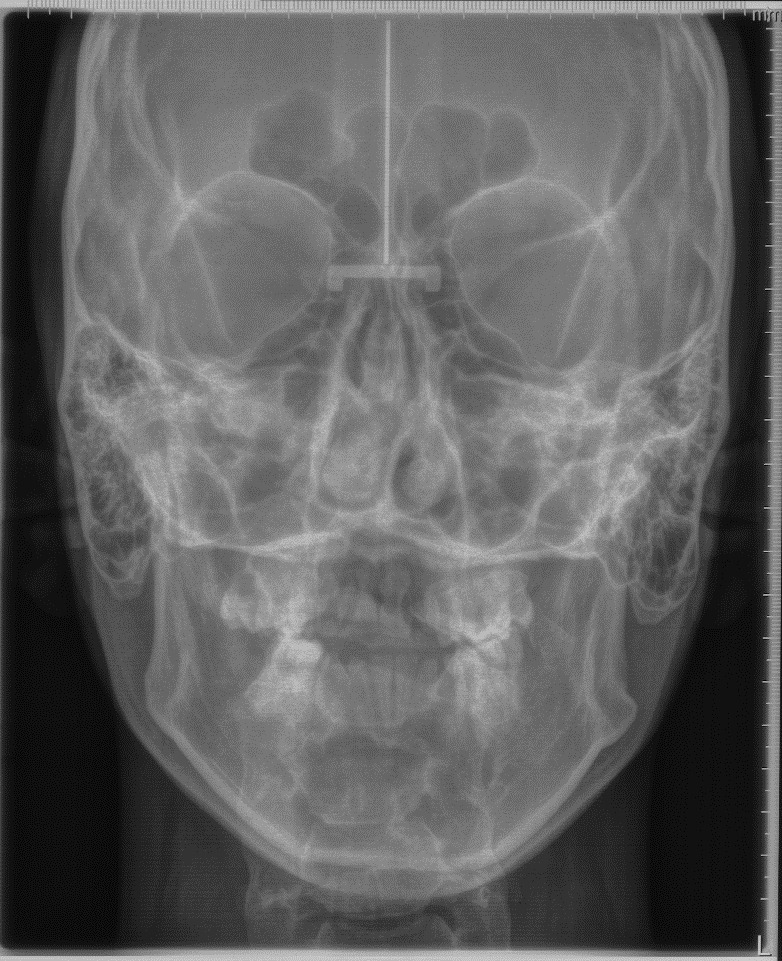
Pre-treatment posterior anterior (P-A) view radiograph of the head.

**Figure 7:**
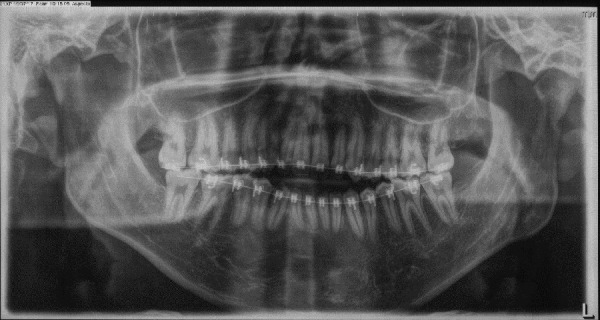
One-year panoramic radiograph follow up.

**Figure 8:**
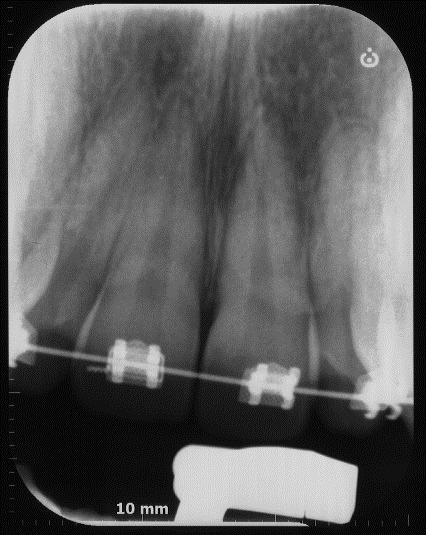
One year follow up periapical radiograph.

A 20-year-old male with a skeletal malocclusion class II with an anterior open bite and a hyperdivergent growth pattern (Figure 3-6) was referred by his orthodontist to a radiology center in Medellín, Colombia. The remitter instructions were to “evaluate probability of ankylosis, because after a year in orthodontic treatment the central maxillary incisor isn’t responding well to the therapy” (Figure 7, 8).

During anamnesis no hereditary conditions were reported by the patient or relatives. Clinical examination the individual revealed a full permanent dentition, misalignment of tooth 1.1, oronasal respiration, lip incompetence, and hypotonic musculature with a 5 mm labial gap; the maximum aperture was 48 mm, with a slight deviation of the mandible to the left.

**Figure 9a:**
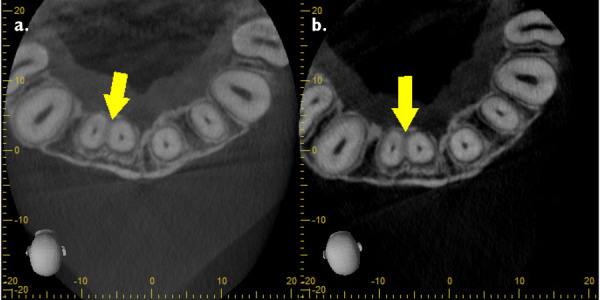
Axial cut denoting union of teeth. **9b.** Short-scale contrast.

**Figure 10a:**
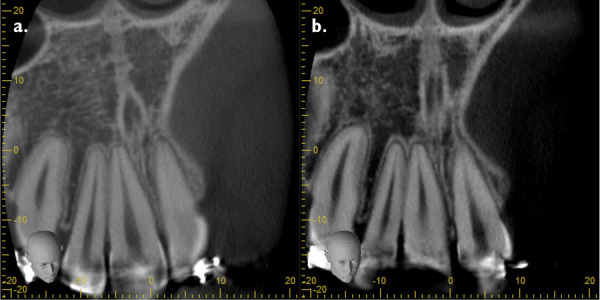
Coronal cut denoting union of teeth. **10b.** Short-scale contrast.

**Figure 11:**
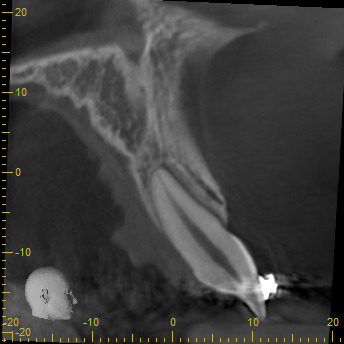
Sagital cut of central incisor

**Figure 12:**
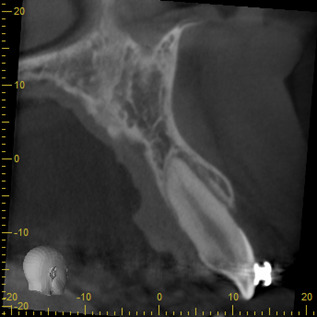
Sagital cut of lateral incisor.

**Figure 13:**
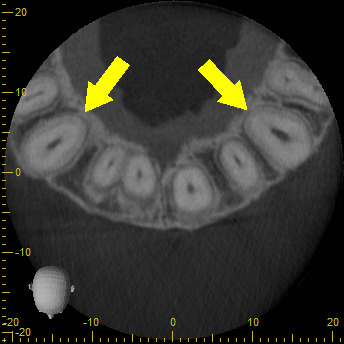
Axial cut of maxillary canines.

**Figure 14:**
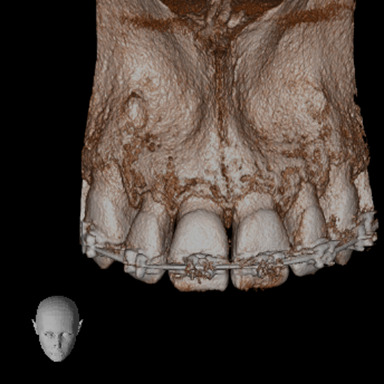
frontal view of 3D reconstruction.

**Figure 15:**
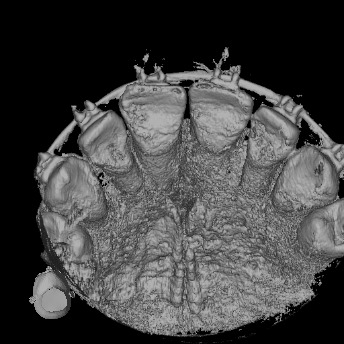
Oclusal view of 3D reconstruction.

A CBCT was made in the central maxillary incisor with an FOV of 40 x 40 mm, the voxel size was set to 125 µm, exposition factors were set to 80 kVp and 7 mA, and the image was obtained using a Veraviewepocs 3D (Jmorita, Osaka, Japan). The final tomographic volume was evaluated by an oral and maxillofacial radiologist with more of 10 years of experience. The following findings were observed: root proximity with a lack of continuation of the periodontal ligament space mesial to the 1.2 tooth and distal to the 1.1 tooth; root union between these teeth near the apex, with no observable division between dental cement tissue and; a palatal root position of the teeth 2.1 and 2.2. Additionally, teeth 1.3 and 2.3 presented radiographic signs of hypercementosis. The findings and signs observed in this volume are compatible with dental concrescence diagnosis (Figure 9 -15). 

**Figure 16:**
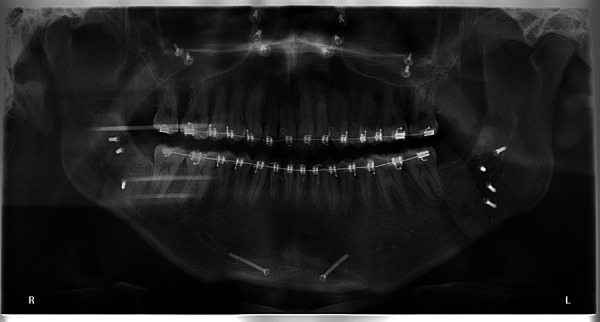
Post-surgery panoramic radiograph.

**Figure 17:**
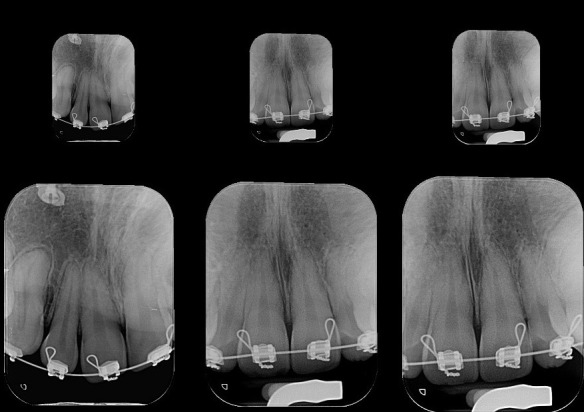
Post-surgery PSP digital periapical films.

**Figure 18:**
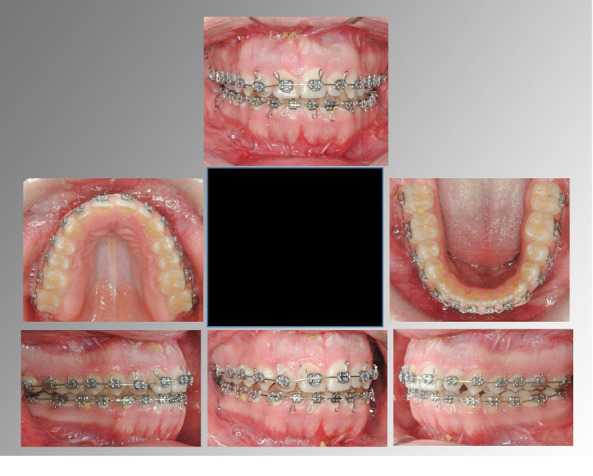
Post-surgery intraoral photographs.

Radiographic diagnosis was obtained and dental ankylosis was discarded. A new treatment plan was instituted according to the findings of the CBCT. Originally, this case had planned to go through orthognathic surgery to correct the class II skeletal malocclusion; however, the option for a dental sectioning procedure was given to the patient. After reading the informed consent, the patient decided not to undergo the dental resection procedure. The orthognathic surgery consisted of modifying the following bones: maxillary, mandible and menton. A maxillary impaction and a sagittal ramus osteotomy procedure were applied to correct the vertical and sagittal component. Mentoplasty surgery was carried out to improve the facial esthetics of the patient. The fixation method used was titanium screws (Figure 16-18); tooth alignment of 1.1 and 1.2 was accomplished with restorative camouflage.

## DISCUSSION

Dental alterations and maxillary formation alteration can occur during the growth and development of an individual ^1^. The correct use of properly selected diagnostic auxiliaries such as tomography can lead to a better understanding of why a patient is not responding as expected to a particular therapy ^11^. In this case report we emphasize the use of bidimensional and tridimensional diagnostic auxiliaries, both at the beginning and during the orthodontic treatment.

For this particular case, a small FOV CBCT was selected to clarify why tooth 1.1 was not moving. The selected size also provided the best resolution to ensure a proper evaluation of the involved tooth. Although it could be argued than a larger CBCT should have been used for planning the orthognathic surgery, tomographic volumes with larger field of view often sacrifice detail by increasing the voxel size, thereby complicating the diagnosis of small areas such as the periodontal ligament space.

**Figure 19:**
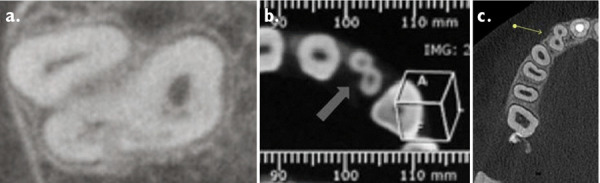
Diferential diagnose images.

Multiplanar reconstruction provided important information for the assessment of the joined teeth. A multiplanar analysis resulted in the following possible diagnoses: dental ankylosis, tooth gemination, tooth fusion and dental concrescence (Figure 19). After a close inspection of the area, dental concrescence was the definitive diagnosis. In this type of dental alteration, it is important to establish whether a tooth sectioning procedure can be applied or not ^12^. This can be done by determining the proximity of the roots and the area in which they are joined together. The larger the area of joining, the less likely a dental sectioning procedure will be viable. 

The application of CBCT volumes is the method preferred by the dental community to determine whether a dental ankylosis is present. Nevertheless, one should consider the reports of false positive results for this tool; therefore, it should be used with caution ^13^. Usually, dental gemination and fusion are clearly observable by x rays due to the union of the roots and/or the crowns. Dental gemination has been reported to be an unlikely situation, with an incidence that ranges from 0.1% to 1% ^14^; therefore, both of these possible outcomes were discarded. Regarding dental ankyloses, the presence of an uninterrupted periodontal ligament space is usually a sign for discarding this type of alteration.

Dental fusion is the union to two or more dental structures in the dentine or enamel tissue, resulting in a united structure that can be recognized by counting the number of dental structures in a full adult dentition. In this case report, this possible diagnosis was discarded by observing the presence of two well-defined crowns, and the union of the teeth was in an apical level.

The incidence of unilateral double teeth is approximately 0.2% in a full adult dentition ^15^. In this case report, the union of the root structures was only by the cement tissue, thus a dental concrescence in the anterior region was obtained ^16^. Ono *et al*. ^17^ used CBCT, microtomography and histopathologic analysis to evaluate dental concrescence between the second and third maxillary molars. The images showed that the union was restricted to dental cement tissue without the involvement of dentin tissue.

Conventional radiographs, such as digital panoramic images, are the most often used and useful method to identify dental anomalies ^18^; Nevertheless, periapical images are often required to complement the diagnosis. However, this approach is often not sufficient to establish a diagnosis, so the use of CBCT is also required in some cases, as presented here ^8^^,^^14^. The use of this imaging technique requires proper justification due to the higher radiation dose compared to the bidimensional approach ^19^.

The treatment of dental anomalies often requires surgery approaches that include dental extraction and dental sectioning. Orthodontic treatment is often necessary to correct function and esthetics, but in cases where the tooth misalignment is not as severe, a restorative approach should be considered due to its less invasive treatment plan ^7^^,^^15^.

## CONCLUSION

Clinical examination complemented by a complete radiographic evaluation is needed prior to the initiation of orthodontic therapy. CBCT technology is a diagnostic tool with important advantages. It provides precision in the anatomical and pathological details that are often difficult to observe in bidimensional images. The correct interpretation of radiographic records contributes to a correct diagnosis, and a multidisciplinary planning approach is necessary to achieve the best possible outcome.

## References

[1] Novoa RU (2015). Radiología de las anomalías dentarias.

[2] Venugopal S, Smitha BV, Saurabh SP (2017). Paramolar concrescence and periodontitis. J Indian Soc Periodontol.

[3] Gunduz K, Sumer M, Sumer AP, Gunhan O (2006). Concrescence of a mandibular third molar and a supernumerary fourth molar report of a rare case. Br Dent J.

[4] Palermo D, Davies-House A (2016). Unusual finding of concrescence. BMJ Case Rep.

[5] Romito LM (2004). Concrescence report of a rare case. Oral Surg Oral Med Oral Pathol Oral Radiol Endod.

[6] Foran D, Komabayashi T, Lin L.M (2012). "Concrescence of permanent maxillary second and third molars: case report of non-surgical root canal treatment. J Oral Sci.

[7] Stanford ND, Hosni S, Morris T (2017). Orthodontic management of a dental concrescence a case report. J Orthod.

[8] Syed AZ, Alluri LC, Mallela D, Frazee T (2016). Concrescence: cone-beam computed tomography imaging perspective. Case Rep Dent.

[9] Schulz M, Reichart PA, Stich H, Lussi A, Bornstein MM (2009). Bilateral malformation of maxillary third molars. Oral Surg Oral Med Oral Pathol Oral Radiol Endod.

[10] Koszowski R, Waskowska J, Kucharski G, Smieszek-Wilczewska J (2014). Double teeth evaluation of 10-years of clinical material. Cent Eur J Med.

[11] Guttal KS, Naikmasur VG, Bhargava P, Bathi RJ (2012). Frequency of developmental dental anomalies in the Indian population. Eur J Dent.

[12] Noar JH, Pabari S (2013). Cone beam computed tomography-current understanding and evidence for its orthodontic applications. J Orthod.

[13] Ducommun F, Bornstein MM, Bosshardt D, Katsaros C, Dula K (2018). Diagnosis of tooth ankylosis using panoramic views, cone beam computed tomography, and histological data a retrospective observational case series study. Eur J Orthod.

[14] Romano N, Souza-Flamini LE, Mendonca IL, Silva RG, Cruz-Filho AM (2016). Geminated maxillary lateral incisor with two root canals. Case Rep Dent.

[15] Castro IO, Estrela C, Souza VR, Lopes LG, De Souza JB (2014). Unilateral fusion of maxillary lateral incisor: diagnosis using cone beam computed tomography. Case Rep Dent.

[16] Hernández-Guisado JM, Torres-Lagares D, Infante-Cossío P, Gutiérrez-Pérez JL (2022). Geminación dental presentación de un caso. Medicina Oral.

[17] Ono M, Shimizu O, Ueda K, Hashimoto J, Shiratsuchi H, Yonehara Y, Honda K (2010). A case of true concrescence diagnosed with cone-beam CT and in vivo micro-CT. Oral Radiol.

[18] Bilge NH, Yesiltepe S, Torenek Agirman K, Caglayan F, Bilge OM (2018). Investigation of prevalence of dental anomalies by using digital panoramic radiographs. Folia Morphologica.

[19] European Commission, Directorate-General for Energy (2011). Cone beam CT for dental and maxillofacial radiolog. Evidence based guidelines.

